# Living Donor Training During ASTS Fellowship: Disparities in Autonomy Between Kidney and Liver Donation. A National Survey

**DOI:** 10.1111/ctr.70671

**Published:** 2026-09-09

**Authors:** Michele Finotti, Silvio Nadalin, Giuliano Testa

**Affiliations:** ^1^ Department of General Visceral and Transplant Surgery University Hospital Tübingen Germany; ^2^ Department of Precision and Regenerative Medicine and Jonian Area (DiMePRe‐J) University of Bari ‘Aldo Moro’ Bari Italy; ^3^ Division of Abdominal Transplantation Annette C. and Harold C. Simmons Transplant Institute Baylor University Medical Center Dallas Texas USA

**Keywords:** autonomy, curriculum, donation, hepatectomy, liver transplantation, living donor, medicine, surgery, transplantation

## Abstract

**Background:**

Living donor transplantation should be a cornerstone for kidney and liver transplant surgeons. However, achieving technical independence, especially in living donor liver transplantation (LDLT), remains a challenge. This survey evaluates the experiences and barriers faced during the American Society of Transplant Surgeons (ASTS) Fellowship Program and proposes solutions to improve autonomy in living donor transplantation.

**Methods:**

A 93‐item questionnaire was distributed via LimeSurvey to former ASTS Fellows, gathering demographic information, training experiences, and autonomy levels in living donor kidney (LDKT) and liver transplantation (LDLT) after the two‐year fellowship. Responses were received from 95 participants. We performed descriptive analyses and exploratory paired comparisons between LDKT and LDLT domains.

**Results:**

A total of 95 former fellows initiated the survey; 41 (43.6%) completed all items and were included in the analyses. The survey revealed significant differences in independence levels between LDKT and LDLT. While 82.9% of fellows felt very or completely confident in LDKT, only 29.2% felt similarly for LDLT (*p* < 0.001). A majority (90.2%) believed the ASTS fellowship structure enabled independence in LDKT, but only 9.7% thought the same for LDLT (*p* < 0.001). Robust training in LDKT was reported, with 63.4% of fellows achieving independence in donor nephrectomy, compared to only 14.6% in donor hepatectomy (*p* < 0.001). Fellows performed a median of 30 living donor nephrectomies as primary surgeons, but only 4 hepatectomies during the fellowship (*p* < 0.0001). Low LDLT case volumes, lack of structured curricula, and limited opportunities to act as primary surgeons were key barriers. 68.3% felt fully or mostly prepared to lead a living donor programme in LDKT, compared to 7.3% in LDLT (*p* < 0.0001).

**Conclusions:**

The ASTS fellowship provides an excellent foundation for LDKT, fostering independence in laparoscopic and robotic approaches. However, independence in LDLT remains difficult to achieve under the current structure. Solutions include formalized curricula, mandated primary surgeon roles in LDLT procedures, and double‐console robotic systems for guided learning. Addressing these gaps is essential to prepare future transplant surgeons for the increasing demands of living donor transplantation programs.

## 1 Introduction

The utilization of living donor transplantation remains a critical strategy to expand access to organ replacement, reduce waiting times, and enhance outcomes for selected recipients [1, 2].

While living donor kidney transplantation (LDKT) is widely adopted and procedurally standardized [3], living donor liver transplantation (LDLT) is performed in fewer centers and demands a higher level of technical proficiency, donor–recipient selection, and robust program infrastructure [3]. In the United States, transplant surgery fellowships have been progressively formalized since the 1990s, with the ASTS fellowship becoming the main structured training pathway over the past decades; however, exposure, supervision, and graded autonomy in living donation can vary substantially across programs [4].

This heterogeneity raises a central educational question: does current fellowship training reliably develop the competencies (technical, cognitive, and organizational) needed to perform living donor procedures safely and to lead or expand living donor programs after graduation?

Concurrently, training modalities are evolving. Simulation (physical, 3D, and video‐based), competency‐based assessment, and dual‐console robotics offer structured opportunities for stepwise skill acquisition, objective feedback, and safer entrustment. But it remains unclear how consistently these tools are integrated into ASTS training and whether they translate into perceived preparedness and independent practice, particularly for LDLT [5, 6, 7, 8].

In order to address these gaps, we conducted a cross‐sectional survey of surgeons who completed an ASTS fellowship, evaluating their exposure to living donation during training, current practice patterns in living donation, perceived preparedness and autonomy across key steps of LDKT and LDLT, and perceived barriers and educational needs. By contrasting LDKT and LDLT within the same respondents, we aimed to identify actionable training targets and to inform a pragmatic roadmap—curriculum standardization, simulation, and robotic dual‐console integration—to accelerate safe competency development in living donor surgery.

## 2 Methods

**FIGURE 1:**
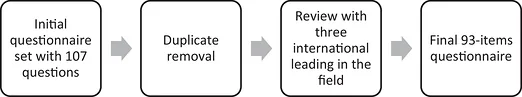
Flow diagram of questionnaire development.

An initial pool of 107 multiple‐choice items was generated from the literature and expert experience. Following the elimination of duplicate and overlapping items and content review by three international experts, the final instrument comprised 93 items (see Figure 1).

The questionnaire was divided into three sections. Firstly, the general population is considered, followed by a focus on living donor liver transplant (LDLT) and the living donor kidney transplant (LDKT) groups. In each section of the study, the following aspects were investigated: donor preoperative evaluation, intraoperative experience and autonomy, postoperative management, perceptions/self‐assessment, and implementation contributions. Intraoperative autonomy was measured using a 4‐point scale (no independence → complete autonomy).

A formal request was submitted to the American Society of Transplant Surgeons (ASTS), resulting in the acquisition of the contact list of former fellows who had successfully completed an ASTS‐accredited fellowship. The finalized survey (LimeSurvey) was distributed via email in August 2024. Participation was voluntary, anonymous, and uncompensated. Each participant was permitted to submit a single response; after submission, answers could not be edited. To reduce accidental partial submissions, participants were required to scroll through all items before submitting. The survey remained open for 8 weeks with two electronic reminders.

The survey collected no identifying information; participation implied consent. The study was conducted in accordance with the Declaration of Helsinki.

### 2.1 Statistical Analysis

Analyses were primarily descriptive. Continuous variables are presented as mean ± SD or median (IQR), as appropriate; categorical variables as counts and percentages. Comparisons between LDKT and LDLT responses within the same participants were considered exploratory and were performed using paired tests. Two‐sided *p*‐values < 0.05 were considered statistically significant for exploratory comparisons; no adjustment for multiple comparisons was applied, and results should be interpreted accordingly. Of 95 respondents who initiated the survey, 41 completed all items and constituted the analytic cohort. Only fully completed surveys were included in paired analyses to preserve internal consistency. For item‐level analyses, denominators vary due to missing responses; percentages are reported over respondents to each item (n shown).

Data were exported from LimeSurvey and analyzed.

## 3 Results

A total of 95 former Fellows initiated the survey; 41 (43.6%) completed all items and were included in the analyses (see Table 1).

**TABLE 1:** Demographics and current employment.

Variable	Results
**Total participants – total respondents**:	95 participants – 41 respondents (43.6%)
**Sex**	29 M (70.7%) 12 F (29.2%)
**Current Age (Median)**	48
**Current Job**	Transplant and HPB surgeon 22 (53.6%) Transplant Surgeon 16 (39%)
**Living donation current main focus**	No 28 (68.2%) Yes 11 (26.8%)
**Currently performing living donor surgery as primary surgeon**	Yes, only liver 3 (7.3%) Yes, only kidney 14 (34.1% Yes, both 9 (21.9%) No 11 (26.8%)

**FIGURE 2:**
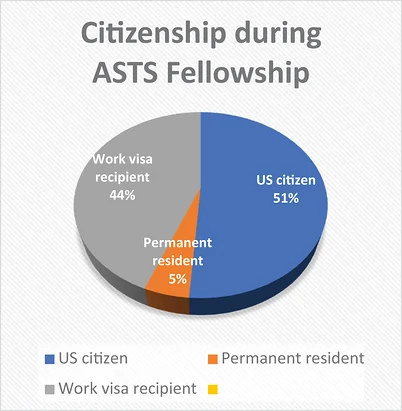
Citizenship status of respondents during ASTS fellowship.

**FIGURE 3:**
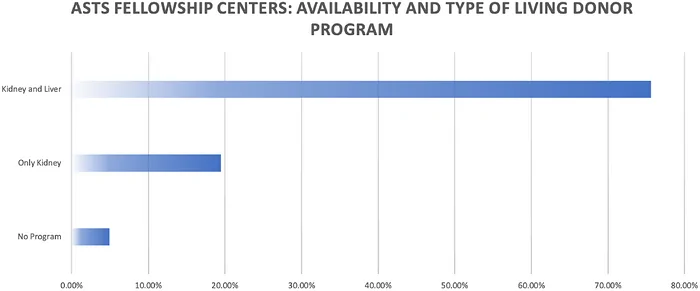
Exposure to living donor programmes during ASTS fellowship. Most fellows (95.1%) trained in centres with living donation programmes, including kidney‐only programmes (19.5%) and combined kidney and liver programmes (75.6%).

Notably, 24.3% of respondents currently practice outside the United States (Canada, Saudi Arabia, Mexico, or Europe). At the time of the ASTS Fellowship, 51.2% were US Citizens, while the other 48.8% were permanent residents or on Visa (Figure 2). Currently, most of the former Fellows are working as a transplant and hepatobiliary surgeon (53.6%) or only transplant surgeon (39%), mainly in an academic setting (43.9%). Most of the Fellows (60.9%) did not have any additional training then residency before the ASTS fellowship, while the others had previous experience in Vascular surgery fellowship, General, Oncological or Trauma surgery. 95.1% of surgeons performed their ASTS fellowship in a center with a living donation program, 19.5% with only kidney living donors and 75.6% with kidney and liver living donor program (Figure 3). In their current practice, 68.2% of respondents did not identify living donation as their primary clinical focus, although 70.7% reported currently performing living donor procedures as primary surgeon.

### 3.1 Kidney Living Donation Curriculum

The median number of living donor kidney transplants performed at participating centres during the ASTS fellowship period was 60 (centre‐level volume, not necessarily cases performed by the fellow). Regarding the training that fellows received during the two‐year ASTS fellowship, the majority of surgeons reported direct involvement in evaluating living kidney donors (73.1%) and participating in the surgical pathway for living donation (90.2%), with a median of 21 donors evaluated per year. The majority of institutions (85.3%) had a clear protocol for defining the inclusion and exclusion criteria for potential living kidney donors, and most commonly held dedicated multidisciplinary meetings on a weekly basis (70.7%). However, the majority of fellows were not directly involved in presenting donors at these meetings (63.4%).

With regard to decision‐making in the preoperative donor evaluation, most fellows (58.4%) required approval for most decisions, whereas 14.6% reported complete autonomy. Regarding imaging assessments, 26.8% of respondents reported having complete autonomy in determining the indications or contraindications for donation. Nevertheless, the majority felt competent in interpreting donor abdominal CT scans (87.6%) and assessing vascular and ureteral contraindications (87.6%).

In terms of operative exposure, minimally invasive approaches were widely used at the training centres, with laparoscopic approaches accounting for 75.6% of cases and robotic approaches accounting for 9.7%. The open approach was used less frequently. During the two‐year fellowship, fellows reported an average of 50 living donor procedures observed and 30 performed as the primary surgeon. The highest autonomy was reported for kidney mobilisation, graft extraction and ureteral mobilisation (85%), followed by renal hilum dissection (65.8%). Overall, 92.6% of fellows reported good autonomy for back‐table kidney preparation. Overall, 53.66% of fellows reported moderate/high autonomy for right donor nephrectomy, rising to 68.29% for left donor nephrectomy. Postoperative management was largely independent (97.56%), typically involving defined protocols (87.8%), although only 39.02% reported involvement in post‐discharge or long‐term follow‐up.

Most surgeons described having complete autonomy when managing postoperative complications in donors, only escalating to the attending surgeon in emergencies (58.5%). When asked about their readiness to start a new living kidney donor programme upon completing their training, most respondents felt fully or mostly prepared (68.3%). Interest in LDKT increased from before to after the fellowship (from 73.1% to 80.5%). However, the majority (87.8%) did not consider an additional third year focused on living kidney donation to be necessary.

Perceptions mirrored this experience: 75.61% felt prepared to select living kidney donors, 82.9% felt ready to perform LDKT independently, and 90.2% felt prepared to recognise and manage postoperative care for living donor kidney transplant recipients. Overall, 80.4% reported that the skills and techniques acquired during their fellowship had enabled them to work independently in LDKT. When asked specifically whether independence in LDKT is a realistic goal under the current ASTS structure, 90.2% agreed, and the same proportion felt that the current structure adequately supports competency in LDKT. (see Table 2).

**TABLE 2:** Training characteristics, and perceived competency in living donor transplantation among survey respondents.

Living Donation curriculum		
	Kidney Living Donation curriculum	Liver Living Donation curriculum
Involved in the selection and evaluation of living donors	Yes 30 (73.1%)	Yes 22 (53.6%)
Clear protocol outlining the inclusion/exclusion criteria for evaluating potential living donors	Yes 35 (85.3%)	Yes 31 (75.6%)
independence in evaluating and clearing patient to donate for living donation	Complete autonomy 6 (14.6%)	Complete autonomy 4 (9.7%)
dedicated meeting for living donation	Yes 29 (70.7%)	Yes 24 (58.5%)
Fellow presenting the patient at living donor meeting	No 26 (63.4%)	No 27 (65.8%)
able to assess the radiological imaging defining indication or contraindication for living donation?	Complete autonomy 11 (26.8%)	Complete autonomy 10 (24.3%)
Ability rate to read and interpret a donor abdominal CT scan for living donation from 1 to 10 (≥7)	36 (87.6%)	25 (60.9%)
Approximate number of living donors per year performed by the center (Median)	60	8
involved in the surgical process for living donation	Yes 37 (90.2%)	Yes 28 (68.2%)
living transplant procedures observed (Median)Number of living donors performed during the fellowship (median)	as primary surgeon 30	as primary surgeon 4
as second assistant 21.5	as second assistant 12
as third assistant 0	as third assistant 5
surgical step in which the fellow has a good level of autonomy	Kidney mobilization 35 (85.3%)	Liver mobilization 35 (85.3%)
	Ureteral Mobilization 35 (85.3%)	Hilar dissection 27 (65.8%)
	Kidney Hilum dissection 27 (65.8%)	Biliary transection 14 (34.1%)
	Graft extraction 33 (80.4%)	Artery transection 20 (48.7%)
		Portal transection 20 (48.7%)
		Parenchymal transection 14 (34.1%)
		Graft extraction 16 (39%)
approach used	Open 13 (14.6%) Minimal Invasive 35 (85.37%)	Open 35 (85.3%) Minimal Invasive 4 (9.7%)
Surgical Independency Perception (≥7, moderate high)	Performing right nephrectomy 22 (53.6%)	performing right hepatectomy 8 (19.5%)
	Performing left nephrectomy 28 (68.2%)	performing left hepatectomy 6 (14.6%)
	Back table preparation 38 (92.6%)	Back table preparation 17 (41.4%)
Feel ready to start a new kidney program on your own in a new institute?	Fully or mostly prepared 28 (68.3%)	Fully or mostly prepared 3 (7.3%)
interest in LDKT (≥7, moderate high)	before starting the ASTS fellowship 26 (73.1%)	before starting the ASTS fellowship 19 (46.3%)
	after completing the ASTS fellowship 33 (80.5%)	after completing the ASTS fellowship 26 (63.4%)
Quality of ASTS education (congress, seminar, webinar, website) (≥7)	Moderate/high 24 (58.5%)	Moderate/high 12 (29.2%)
completed a third year specifically focused on living donation	No 36 (87.8%)	No 32 (78%)
Perceived independency (Fully‐mostly prepared or independent)	independently perform living donor kidney transplants 34 (82.9%)	independently perform living donor liver transplants 12 (29.2%)
	recognize and manage postoperative care for living donor kidney transplant recipients 37 (90.2%)	recognize and manage postoperative care for living donor liver transplant recipients 19 (46.3%)
	In donor nephrectomy 26 (63.4%)	In donor nephrectomy 6 (14.6%)
	performing kidney recipient from living donation 38 (92.6%)	performing liver recipient from living donation 12 (29.2%)
In which stages of living donor procedures do you feel you have the most autonomy	Donor preparation 25 (60.9%)	Liver mobilization 30 (73.1%)
	Kidney Donor Nephrectomy 23 (56.1%)	Hilum dissection 26 (63.4%)
	Implantation 37 (90.2%)	Liver transection 13 (31.7%)
	Donor follow‐up 27 (65.8%)	Able to determinate site of vascular transection 14 (34.1%)
	Recipient follow‐up 35 (85.3%)	Able to determinate site of biliary transection 12 (29.2%)
	I did not have autonomy 2 (4.8%)	Standard back table 26 (63.4%)
		Back table with vascular reconstruction 17 (41.4%)
		Standard living donor implantation 23 (56.1%)
		Living donor implantation with vascular reconstruction 12 (29.2%)
		I did not have autonomy 4 (9.7%)
How confident are you that the skills and techniques acquired during ASTS will allow you to be independent in living donor	Confident – Completely confident 31 (80.4%)	Confident – Completely confident 18 (43.9%)
Is becoming independent in living donor transplantation a realistic goal after completing the ASTS fellowship	As currently structured Agree/extremely agree 37 (90.2%)	As currently structured Agree/extremely agree 4 (9.7%)
	After potential changes Agree/extremely agree 33 (80.4%)	After potential changes Agree/extremely agree 6 (14.6%)
Do you agree the current fellowship training structure adequately supports the competency of surgeons performing living donor transplantation?	Agree/extremely agree 37 (90.2%)	Agree/extremely agree 5 (12.2%)
What do you perceive as the main barrier/ barriers for fellows to receive comprehensive education in living and recipient transplantation?	Attending surgeons not confident enough to allow fellows to perform the surgery 17 (41.4%)	Attending surgeons not confident enough to allow fellows to perform the surgery 27 (65.8%)
	Not enough experience in pre and/or post operative donor management 5 (12.2%)	Not enough experience in pre and/or post operative donor management 7 (17%)
	Not enough surgical exposure 14 (34.1%)	Not enough surgical exposure 26 (63.4%)
	Other attending surgeons are learning LDKT 10 (24.3%)	Other attending surgeons are learning LDLT 28 (38.2%)
	Institute needs to demonstrate good outcomes 17 (41.4%)	Institute needs to demonstrate good outcomes 28 (38.2%)
	Low volume of the center 18 (43.9%)	Low volume of the center 34 (82.9%)
	Lack of personal interest 9 (21.9%)	Lack of personal interest 4 (9.7%)
Would you limit the training only in high volume center in living donation	Yes 17 (41.4%)	Yes 24 (58.5%)

### 3.2 Liver Living Donation Curriculum

The median number of living donor liver transplants performed at participating centres was eight per year. During the two‐year ASTS fellowship, 53.6% of surgeons reported being directly involved in evaluating living liver donors, and 68.2% participated in the surgical pathway. On average, they evaluated 21 donors per year. While most institutions (75.6%) had defined protocols for donor selection and held dedicated multidisciplinary meetings, most commonly on a weekly basis (58.5%), most fellows were not involved in presenting donors (65.8%).

Upon completing their fellowship, the majority of fellows reported a lack of independence when it came to evaluating and clearing donors for LDLT: 53.6% required attending approval for all decisions, while only 9.76% reported complete autonomy. Regarding imaging assessment, 24.3% reported complete autonomy in defining indications and contraindications. Low case volume and limited fellow involvement were frequently cited as reasons for gaps in donor selection skills.

In terms of surgical approach, most centres used an open approach (85.4%), whereas minimally invasive approaches were less common (laparoscopic 4.3%, robotic 4.3%). Most fellows (78%) did not pursue a third year specifically focused on LDLT. During the fellowship, the median number of living donor liver procedures observed (in fellows where living liver donation was available) was six, with four performed as the primary surgeon. Reported autonomy was higher for liver mobilisation (85.3%), hilar dissection (65.8%) and arterial/portal transection (48.7%), and lower for biliary transection and parenchymal transection (34.1%). Insufficient independence when performing right and left donor hepatectomy was common (60.8% and 60.9% respectively), with only 7.3% and 4.8% of fellows reporting full independence in these procedures. Autonomy in back‐table liver preparation was reported by 48.6% of fellows.

In terms of programme leadership, the majority of respondents felt that they were not at all or only slightly prepared to start a new LDLT programme upon completing their fellowship (31.7% and 24.3%, respectively). Interest in LDLT increased during training (46.3% reported high interest before the fellowship, compared to 68.4% after). In terms of perceived preparedness, 48.78% of respondents felt prepared to select living liver donors, 46.3% felt prepared to recognise and manage postoperative care for LDLT recipients, and 34.1% felt prepared to perform donor liver retrieval with the assistance of an expert surgeon. Furthermore, 31.7% of respondents reported non‐independence. Regarding the recipient operation after living donation, 29.2% reported independence or mostly independence; 26.8% reported independence only in standard cases with classical anatomy; 14.6% reported independence only with expert assistance; and 12.2% reported non‐independence. When asked whether independence in LDLT is a realistic goal under the current ASTS structure, 75.6% disagreed. Even with potential changes to ASTS training, 65.8% still considered achieving independence in LDLT by the end of the fellowship unrealistic.

The majority (56.1%) felt that the current liver transplant training structure does not adequately support competency in LDLT. While a majority (68.3%) agreed that a formalised LDLT surgical training programme could enhance skills by the end of a fellowship, only 36.59% considered excellent LDLT training to be essential for all transplant surgeons. Respondents emphasised the importance of HPB training (70.73%) and experience in higher‐volume countries (73.17%), but only 35.5% considered an additional year of training to be a viable solution. (see Table 2).

### 3.3 Comparison between LDKT and LDLT

The survey revealed significant differences in perceived independence. 82.9% of fellows felt very or completely confident in LDKT, compared with 29.2% for LDLT (*p* < 0.001). The majority (90.2%) believed that the current ASTS structure enables independence in LDKT, compared to 9.7% for LDLT (*p* < 0.001). Fellows reported independence in the donor operation in 63.41% of cases for donor nephrectomy versus 14.64% for donor hepatectomy (*p* < 0.001). Fellows performed a median of 30 living donor nephrectomies as the primary surgeon, but only four hepatectomies (*p* < 0.0001). The most frequently cited barriers were low LDLT volume, lack of structured curricula, and limited opportunities to act as primary surgeon. The readiness to lead a living donor programme was 68.3% for LDKT versus 7.3% for LDLT (*p* < 0.0001).

### 3.4 Comparison between Low‐Volume and High‐Volume Living Donor Centers

An exploratory, volume‐based subanalysis was performed for both LDLT and LDKT. A total of 39.0% of respondents were trained in high‐volume LDLT centres (defined as centres performing ≥10 LDLTs), with a median centre volume of 20 LDLTs per year.

Respondents who were trained in high‐volume LDLT centres reported greater operative exposure and a higher level of perceived autonomy in several technical steps of LDLT. No respondent from the low‐volume group reported performing donor hepatectomy as the primary surgeon during their fellowship. In contrast, the mean number of donor hepatectomies performed by respondents from the high‐volume group as the primary surgeon was 14.4. Respondents from high‐volume centres were significantly more likely to have autonomy in liver transection than those from low‐volume centres (62.5% vs 18.2%, *p* = 0.047). Independent or mostly independent performance of donor hepatectomy was also more frequent in the high‐volume group (37.5% vs 0%), though this difference was not statistically significant (*p* = 0.057). Despite these favourable results, fewer than half of the respondents from high‐volume LDLT centres felt fully or mostly prepared to start a new LDLT programme, and only a minority considered independent LDLT practice to be a realistic goal following completion of the current ASTS fellowship programme.

Of the total cohort, 26.8% trained in high‐volume LDKT centres (defined as centres performing ≥50 LDKT procedures per year), with a median centre volume of 80 LDKT procedures per year.

Respondents who were trained in high‐volume LDKT centres reported greater active operative exposure, performing a median of 40 donor nephrectomies as the primary surgeon, compared to 10 in low‐volume centres. Training in a high‐volume centre was also associated with a higher perceived autonomy in key technical steps of donor nephrectomy, including renal hilum dissection (90.9% vs 43.8%, *p* = 0.018), autonomy in right donor nephrectomy ≥7/10 (81.8% vs 37.5%, *p* = 0.047), and autonomy in the kidney donor nephrectomy phase (81.8% vs 37.5%, *p* = 0.047). Respondents from high‐volume centres also had greater confidence that their fellowship‐acquired skills would allow them to perform LDKT independently (100% vs 60.0%, *p* = 0.024). However, unlike LDLT, perceived preparedness for LDKT remained high even among respondents from low‐volume centres, and most respondents in both groups considered achieving independence in LDKT a realistic goal upon completing an ASTS fellowship.

## 4 Discussion

In our sample, the majority of respondents completed a fellowship at centres with a living donation programme, and now mainly work in transplant and/or hepatobiliary practice, including outside the United States. An interesting initial finding is that interest in living donation increases during the ASTS fellowship (from 46% before ASTS to 68% after ASTS), suggesting that the training path raises motivation. However, this rarely results in a professional identity centred on living donation. Despite broad exposure (approximately 75% were trained in centres offering both kidney and liver living donation), only some surgeons declare living donation as their primary focus.

This discrepancy between training exposure and stated professional focus suggests that, although the experience gained during the fellowship is extensive and seemingly sufficient, it does not always translate into a professional identity centred on living donation. This is particularly evident in liver donation, where achieving autonomy is challenging during the fellowship (for most participants, this is not a feasible objective within the ASTS fellowship).

This finding is consistent with the results of other recent U.S. surveys. These surveys indicate a high level of interest in expanding LDLT because LDLT is recognised as an effective way of reducing waiting lists and increasing the donor pool. However, a lack of structured fellowship pathways and a shortage of suitably qualified staff remains a key bottleneck, often necessitating the recruitment of experienced professionals from overseas rather than from within the US [9, 10].

A recent multidisciplinary consensus also highlighted that the limited impact of LDLT in Western countries depends partly on the small number of competent surgeons and therefore on the need for robust training pathways with clear competency targets, particularly for rare and complex procedures such as donor hepatectomy [9].

Specifically, reported autonomy is higher in LDKT than in LDLT. In LDKT, autonomy is greater for left nephrectomy than right (68.2% vs. 53.6%), which is likely linked to anatomical factors and greater standardisation and volume of the procedure. In LDLT, achieving full autonomy within the standard two ASTS years remains the exception; the technical complexity of the procedure, its relative rarity and the need to ensure maximum donor safety realistically limit progression to independence. Respondents also report that, even following potential changes to the fellowship structure, achieving full autonomy in LDLT would remain challenging.

The exploratory, volume‐based subanalysis sheds more light on the structural differences between LDLT and LDKT training. For LDLT, training in a high‐volume centre was associated with greater operative exposure and a greater sense of autonomy during technically demanding steps, especially liver transection and donor hepatectomy. However, the findings also show that volume alone is insufficient to ensure independence. Even among respondents trained in high‐volume LDLT centres, fewer than half felt fully or mostly prepared to start a new LDLT programme, and only a minority considered independent LDLT practice to be realistic by the end of the current ASTS fellowship structure. These results imply that LDLT training necessitates not only sufficient case volume, but also deliberate entrustment, graded autonomy, structured curricula, dedicated mentorship and post‐fellowship proctorship.

In contrast, the LDKT subanalysis suggests a more mature and standardised training environment. High‐volume LDKT centres were associated with greater operative exposure, greater autonomy in donor nephrectomy and stronger confidence in achieving independent practice. Nevertheless, preparedness in LDKT remained relatively strong, even among respondents from lower‐volume centres. Most respondents in both LDKT volume groups considered achieving independence after the ASTS fellowship to be realistic, and they perceived the current fellowship structure to be adequate in supporting LDKT competency. This contrasts sharply with LDLT and supports the interpretation that LDKT has become a more standardised, reproducible and broadly teachable component of transplant fellowship training.

### 4.1 Institutional Barriers and the Role of Volume

Our results support recent surveys and consensus statements regarding low LDLT case volume per centre, a long learning curve, the need to demonstrate excellent outcomes and limited staff confidence in granting autonomy to fellows (see Table 3). It is also important to distinguish between technical autonomy and the ability to set up and manage an LDLT programme (including donor/recipient selection, multidisciplinary governance and safety audits), as this requires organisational skills as well as technical ones.

**TABLE 3:** Main barriers for LDLT training during ASTS fellowship.

Main barriers in LDLT
Low volume of the center
Institute needs to demonstrate good outcomes
Other attending surgeons are learning LDLT
Attending surgeons not confident enough to allow fellows to perform the surgery
Not enough surgical exposure

Another finding consistent with current practice is that left hepatectomy is associated with lower independence than right hepatectomy, reflecting the more limited diffusion of left‐lobe donation and, in some scenarios, greater reconstructive complexity.

### 4.2 The Robotic Console as an Educational (and Safety) Lever

Today, we have a unique and important opportunity in transplantation: the robotic technique. Robotics offers two opportunities: it provides a teaching platform and reduces the learning curve for living donor procedures, particularly donor nephrectomy (RDN), and, in selected centres, donor hepatectomy. Meta‐analyses and contemporary studies indicate that the performance of RDN is at least comparable to that of laparoscopy, with a favourable learning pattern and potential ergonomic and visual benefits. Data suggest that the learning curve stabilises with relatively contained volumes when implementation is structured. In short, the learning curve for robotic nephrectomy is usually faster than for laparoscopy, enabling surgeons to become autonomous within the two‐year fellowship programme. The same is true for living donor kidney transplantation (LDKT), because the procedure is simpler than living donor liver transplantation (LDLT) and volumes are usually higher [9, 10, 11, 12].

**FIGURE 4:**
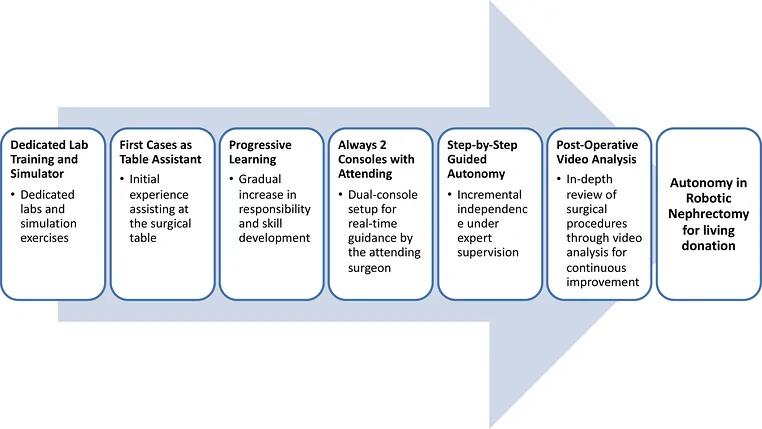
Proposed stepwise training pathway for living donor nephrectomy.

From an educational perspective, the dual console enables synchronous coaching, fine modulation and full delegation of the intervention, as well as immediate tutor takeover. It transforms the operative moment into a reproducible, proficiency‐based training setting, particularly in LDLT (see Figure 4). In LDLT, there is an international consensus that safe expansion requires documented proficiency, proctorship and a gradual approach. Robotics can facilitate this technical scaffolding provided that programmes define case criteria, supervision and competency cut‐offs.

In addition, using 3D models for preoperative rehearsal alongside the robotic console for execution creates continuity between simulation and the operating room (OR). The same ‘gestures’ can be practised ex vivo and then transferred in vivo. Objective metrics (e.g. time taken for critical steps, economy of motion and sentinel errors) can be used to certify progress and identify when the trainee is ready to perform the next part of the procedure.

### 4.3 Educational Implications: What Is Missing Today

When interpreting our results, it is important to note that the ASTS fellowship is not a single, uniform training pathway. In practice, ASTS fellowships can be either transplant‐only or combined with hepatopancreatobiliary (HPB) training. For this reason, the expectations and operative opportunities of fellows differ, and this may directly influence their perceived autonomy upon completing the programme. In kidney transplantation, it is commonly expected that by the end of the fellowship, the trainee will be able to perform both the donor nephrectomy and the kidney implantation with progressive autonomy. In liver transplantation, however, the situation is more heterogeneous: donor hepatectomy may not form part of the surgical training programme in transplant‐only fellowships, and even in centres with LDLT activity, key steps such as living donor back‐table work and/or parts of recipient implantation may be prohibited for fellows due to safety concerns, pressure to achieve good outcomes, and local policies. Therefore, part of the gap we observed between exposure to living donation and achieving LDLT independence is likely explained not only by volume and complexity, but also by structural differences in what fellows are realistically permitted (and expected) to do across ASTS programmes.

From an educational perspective, this means that standardisation should not assume identical goals for all ASTS fellows. A more realistic approach would be to define two competency pathways aligned with the two main fellowship profiles: [1] a core transplant track ensuring competence in living donor kidney transplantation (LDKT) and a solid foundation in living donor liver transplantation (LDLT) principles, including donor evaluation, imaging interpretation, donor safety, recipient management and supervised exposure to critical steps; and [2] an advanced transplant and hepatopancreatobiliary/living donor liver transplantation (HPB/LDLT) track for trainees aiming to become independent living donor liver surgeons. This track would involve a clear minimum exposure, structured graded autonomy and longer, high‐volume‐focused experience, including formal rotations in selected high‐volume LDLT centres. Finally, our data support the idea that a dedicated certification for living donor surgery could be valuable for trainees and hiring institutions alike. Beyond the current regulatory requirements (e.g. UNOS‐related credentialing), an ASTS‐endorsed, competency‐based certificate with separate modules for LDKT and LDLT could give fellows a tangible advantage when applying for jobs, while also creating a shared understanding of what ‘trained’ means for programmes and centres. This credential should be linked to objective milestones such as simulation, structured assessment, graded autonomy and proctorship, rather than simply counting cases, and it could help to reduce variability between transplant‐only and transplant + HPB pathways, making expectations clearer for trainees and faculty.

Our data and the literature outline three urgent training needs. (see Table 4).

1. Standardize the LDLT curriculum Within the ASTS fellowship
2. Define stepwise objectives (e.g. donor/recipient evaluation, resection/reconstruction techniques, management of complications) with autonomy milestones and mandatory proctorship
3. Set minimum exposure requirements (e.g. case mix, complexity, active role), but above all use competency‐based assessments rather than case counts.
4. Clarify what can realistically be achieved in two years in terms of LDLT autonomy. Consider a dedicated third year or rotations in high‐volume centres (including abroad) that are formally integrated into the fellowship.
5. Recognise that full mastery of donor hepatectomy takes several years and requires post‐fellowship mentorship. As our survey also shows, achieving autonomy within the fellowship is not realistic; however, it is essential to define the basic knowledge and skills that every living donation surgeon should have.
6. Make pre‐/intra‐operative teaching widespread With modern tools
  - Tactile 3D simulation, milestone checklists, video scenarios with annotation of sentinel errors, dry/wet labs, and tracking of objective metrics.
  - Use structured rating scales to make decisions on progression of autonomy transparent.
7. Use robotics as a teaching platform
  - Dual console for coaching and graded autonomy with a digital logbook (recording times, complications and readmissions) and certifiable competency thresholds.
  - In LDKT, robotics can accelerate the achievement of safe autonomy within the fellowship. In LDLT, careful case selection and rigorous proctorship can enable it to work as a bridge within pathways.
  - Integrate 3D rehearsal and robotics to create a continuous path from planning to the operating room (OR), improving confidence and safety.

**TABLE 4:** Proposed solutions to improve LDLT training during ASTS fellowship.

Challenges and Proposed Solutions for LDLT Training During ASTS Fellowship	
Training Essentials	HPB training is essential
	Establishment of a formalized surgical training
	Training in other countries with more volume
Training Limitations	Limit LDLT training only in high‐volume centers
	Limit LDLT training only in centers with HPB program

### 4.4 Limitations and Future Directions

The small number of complete responses limits the study's statistical power and exposes it to selection and recall bias. However, the internal and external consistency of the signals (LDKT autonomy > LDLT, increased interest in LDLT, and the central role of volume, curriculum and proctorship) support the validity of the study. Furthermore, this study captures self‐reported preparedness and perceived autonomy, rather than objectively assessed surgical competence. Future prospective multicentre studies should validate LDLT‐specific competency metrics, measure the effect of dual consoles and 3D simulations on learning times and safety, and test the organisational sustainability of proficiency‐based pathways in different settings (academic versus non‐academic).

## 5 Conclusion

The unifying message is educational: there is plenty of interest in LDLT, but not enough has been translated into widespread competence. A standardised curriculum incorporating 3D simulation and the robotic console as key infrastructure, alongside structured proctorship and objective performance assessments, could improve the learning curve, expand the competent workforce and, most importantly, increase access to safe living donation for donors and recipients.

## Funding

This study received no external funding.

## Ethics Statement

This Study Was Deemed Exempt From Irb Review as It Involved Analysis of De‐identified Data

## Conflicts of Interest

The authors declare no relevant conflicts of interest.

## Data Availability

The data that support the findings of this study are available from the corresponding author upon reasonable request.
